# Urodynamik in Deutschland: Indikationen und Anwendung im urologischen Klinik- und Praxisalltag

**DOI:** 10.1007/s00120-025-02579-5

**Published:** 2025-04-14

**Authors:** Viktoria Menzel, Livia Kontschak, Marcus Sondermann, Markus Grabbert, Angelika Borkowetz, Sherif Mehralivand, Nicole Eisenmenger, Johannes Huber, Christian Thomas, Daniela Schultz-Lampel, Martin Baunacke

**Affiliations:** 1 Klinik und Poliklinik für Urologie, TU Dresden, Fetscherstr. 74, 01307 Dresden, Deutschland; 2 Klinik für Urologie, Universitätsklinikum Freiburg, Freiburg, Deutschland; 3 Klinik und Poliklinik für Urologie, Universitätsmedizin Rostock, Rostock, Deutschland; 4 Reimbursement GmbH, Hürth, Deutschland; 5 Klinik für Urologie, Universitätsklinikum Heidelberg, Heidelberg, Deutschland; 6 Kontinenzzentrum Südwest, Schwarzwald-Baar Klinikum, Villingen-Schwenningen, Deutschland

**Keywords:** Kontinenz, Blasenfunktionsstörung, Versorgungsforschung, Epidemiologie, Harninkontinenz, Continence, Bladder dysfunction, Public health, Epidemiology, Urinary inontinence

## Abstract

**Hintergrund:**

Urodynamische Studien (UDS) sind ein etabliertes Diagnostikverfahren in der Urologie. Internationale Studien, auch in Deutschland, zeigen jedoch einen Rückgang ihrer Anwendung. Gründe hierfür könnten Leitlinienanpassungen und veränderte Indikationsstellungen sein.

**Fragestellung:**

Ziel ist es, die Versorgungsrealität und Indikationsverteilung für UDS in urologischen Kliniken und Praxen in Deutschland zu untersuchen.

**Material und Methoden:**

Zwischen 03/2023 und 10/2023 wurden landesweit 259 urologische Kliniken und stichprobenartig 280 niedergelassene Urologen zur UDS befragt. Daten zu angewandten Methoden, durchführendem Personal und Indikationsverteilung wurden erhoben.

**Ergebnisse:**

Es nahmen 80 % der Kliniken und 44 % der Praxen teil. 58 % der Kliniken führen < 100 UDS pro Jahr durch. 15 % der Praxen führen selbstständig UDS durch. Verfahren wie Uroflowmetrien (99 %) und Zystomanometrien (98 %) werden flächendeckend angewandt, während Videourodynamiken (53 %) seltener zur Verfügung stehen. Häufigste Indikationen in Kliniken sind neurogene Blasenfunktionsstörungen (24 %), überaktive Harnblasen (21 %) und Belastungsinkontinenz bei Frauen (19 %). Praxen zeigen ähnliche Schwerpunkte: neurogene Blasenfunktionsstörungen (30 %) und überaktive Harnblasen (26 %). Universitätskliniken und spezialisierte Zentren führen signifikant häufiger UDS bei komplexen Krankheiten durch, während die Versorgung der weiblichen Belastungsinkontinenz häufiger in nichtuniversitären Kliniken erfolgt.

**Schlussfolgerung:**

Trotz sinkender Fallzahlen bleiben UDS zentral für die Diagnostik. Die Indikationsverteilung variiert je nach Kliniktyp. Diese Ergebnisse liefern Einblicke in die Praxisanwendung von UDS in Deutschland und unterstreichen ihre Rolle in der urologischen Diagnostik.

**Graphic abstract:**

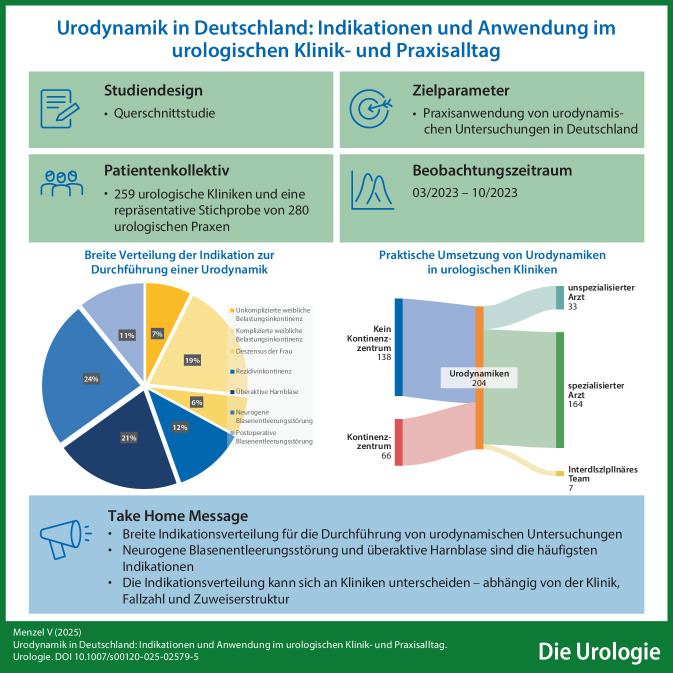

## Abstract (en)

**Background:**

Urodynamic studies (UDS) are an established diagnostic tool in urology. However, international studies, including those from Germany, indicate a decline in their use. This trend may be attributed to guideline adjustments and changes in indications.

**Objective:**

The study aims to analyse the current practice and distribution of indications for UDS in urological clinics and practices in Germany.

**Materials and methods:**

Between March 2023 and October 2023, 259 urological clinics and a representative sample of 280 outpatient urologists nationwide were surveyed about their UDS procedures. Data on methods, personnel and indications were collected.

**Results:**

In all, 80% of clinics and 44% of practices participated. 58% of clinics performed fewer than 100 UDS annually. Only 15% of practices performed UDS themselves. Common procedures such as uroflowmetry (99%) and cystometry (98%) are widely implemented, whereas video urodynamics (53%) are less frequent. The most common indications in clinics are neurogenic bladder dysfunction (24%), overactive bladder (21%) and female stress incontinence (19%). Practices showed similar trends, with neurogenic bladder dysfunction (30%) and overactive bladder (26%) as primary indications. University hospitals and specialized centres performed UDS more often for complex conditions, while non-university clinics frequently addressed stress incontinence.

**Conclusion:**

Despite declining numbers, UDS remain central to urological diagnostics. Indication patterns vary by clinic type. These results provide insights into the application of UDS in Germany and underline its important role in urological diagnostics.

## Einleitung

Die Durchführung urodynamischer Studien (UDS) ist ein etabliertes Verfahren und eine komplexe Diagnostik, die in der Urologie nach wie vor flächendeckend angewendet wird [1, 2]. Um verlässliche Ergebnisse zu erhalten, ist eine hochwertige Technik erforderlich [3]. International orientiert sich die Durchführung einer Urodynamik nach den Vorgaben der „good urodynamic practices“ der Internationalen Kontinenzgesellschaft (ICS; [4, 5]). Die Durchführung von UDS erfordert spezialisierte Ausrüstung und geschultes Personal, um die Genauigkeit und Zuverlässigkeit der Ergebnisse zu gewährleisten [6]. Ziele einer urodynamischen Untersuchung sollten einerseits die Beurteilung der Blasen‑, Detrusor- und Sphinkteraktivität und andererseits die Risikoeinschätzung für den oberen Harntrakt sein [7].

Internationale Studien, darunter 3 aus den USA über einen Zeitraum von 2010 bis 2014 und eine koreanische Studie von 2010 bis 2015, deuten auf einen rückläufigen Trend bei der Nutzung von UDS hin [8, 9]. Auch in Deutschland zeigt sich ein deutlicher Rückgang der Urodynamikverfahren, um etwa −14 % im Zeitraum von 2013 bis 2018. In urologischen Kliniken lag dieser Rückgang sogar bei −45 % von 2013 bis 2019 [10]. Angesichts der sinkenden Zahlen der UDS stellt sich die Frage nach Gründen des Rückgangs. Hier könnten mögliche Indikationsänderungen eine Rolle spielen wie beispielsweise die Anpassung der EAU-Leitlinien zur Harninkontinenz im Jahre 2013. Nachdem in mehreren Studien, wie der VaIUE-Studie (Value of Urodynamic Evaluation) und der VUSIS-2-Studie (Value of Urodynamics before Stress Incontinence Surgery), die Irrelevanz einer UDS zur weiteren Diagnostik bei unkomplizierter Belastungsinkontinenz nachgewiesen werden konnte, erfolgte eine überarbeitete Empfehlung der Indikation zur Durchführung einer UDS [11, 12]. Von der Empfehlung „präoperativ eine urodynamische Untersuchung durchzuführen, wenn Symptome einer überaktiven Harnblase, eine Vorgeschichte für frühere Operationen oder der Verdacht auf Miktionsstörungen vorliegt“ wurde Abstand genommen [13]. Die UPSTREAM-Studie (Urodynamics for Prostate Surgery Trial: Randomized Evaluation of Assessment Methods) untersuchte den Nutzen der UDS zur Indikationsstellung und zeigte, dass sie bei Männern mit LUTS („lower urinary tract symptoms“) in der Routinediagnostik keinen signifikanten Mehrwert erbringt [14].

Letztlich gibt es aber keine Informationen über die Indikationsverteilung und Anwendung der Urodynamik in Deutschland, um die genannten Aspekte in einen Kontext zu setzen. Ziel dieser Arbeit ist es, die aktuelle Versorgungsrealität und die Indikationsstellungen für UDS in deutschen urologischen Kliniken und Praxen umfassend zu analysieren, um Einblicke in die Praxisanwendung und potenzielle Lücken in der Versorgung zu gewinnen.

## Material und Methoden

Im Rahmen dieser Studie wurden urologische Kliniken und ambulante UrologInnen befragt. Es wurde erhoben, welche invasiven urodynamischen Untersuchungen wie Zystomanometrie, Urethradruckprofilmessung und Videourodynamik sowie die nicht-invasive Uroflowmetrie durchgeführt werden. Es erfolgte eine postalische Befragung aller urologischen Kliniken in Deutschland, die im Jahr 2019 UDS durchgeführt haben (*n* = 259). Zur Identifizierung der urologischen Kliniken wurden die Qualitätsberichte deutscher Krankenhäuser unter Verwendung des OPS-Codes (Operationen- und Prozedurenschlüssel) 1‑334 einschließlich der Untercodes genutzt. Urologische Abteilungen wurden anhand des Abteilungscodes (FAB) identifiziert. Die Qualität der Krankenhausqualitätsberichte hängt von der Dokumentation ab, die von dem jeweiligen Krankenhaus bereitgestellt wurde. Die Daten der Qualitätsberichte wurden auf Fehlerhaftigkeit geprüft. Die Qualitätsberichtsdaten wurden mit Hilfe des Analysetool „reimbursement.INFO“ (RI Innovation GmbH, Hürth, Deutschland) ausgewertet.

Die Befragung ambulanter UrologInnen erfolgte anhand einer repräsentativen Stichprobe. Für diese Befragung erfolgte die Erstellung einer Liste aller niedergelassenen UrologInnen nach den Informationen der 17 kassenärztlichen Vereinigungen in Deutschland. Zum Zeitpunkt der Erstellung der Liste waren 3232 UrologInnen gelistet (November 2022). Diese Liste enthält Informationen über die Niederlassungsart (Medizinisches Versorgungszentrum, selbständige UrologInnen) und den Standort (Bundesland und Stadt). Mit diesen Daten erfolgte die Erstellung einer repräsentativen Stichprobe hinsichtlich der Verteilung der Praxisform, des Bundeslandes und dem örtlichen Umfeld (Stadt vs. Land, Einwohnerzahl ≥ 100.000 vs. < 100.000; *n* = 280).

Urologische Kliniken erhielten im Mai 2023 einen Fragebogen und ambulante UrologInnen im Oktober 2023. Nicht-Responder wurden 2 Monate später erneut kontaktiert. Der Fragebogen hat die angewandten Untersuchungsverfahren, die Art der Durchführung und die prozentuale Verteilung der Indikationsstellung zur Durchführung einer UDS abgefragt. Die prozentuale Verteilung der Indikationsstellungen der Kliniken wurden auf deren Urodynamikzahlen entsprechend der Qualitätsberichtsdaten auf absolute Zahlen hochgerechnet. Die prozentuale Verteilung der Indikationsstellungen der ambulanten UrologInnen wurde auf Basis der jährlichen Gesamtzahl urodynamischer Untersuchungen in absolute Zahlen hochgerechnet. Ausgangspunkt hierfür war die Zahl an UDS, welche die Kliniken im Jahr 2019 entsprechend der Qualitätsberichte durchgeführt haben. Weiterhin wurden strukturelle Parameter und Entwicklungen der UDS-Zahlen erhoben. Die Ergebnisse der Auswertung der strukturellen Entwicklung der UDS wurde bereits publiziert [15].

Aufgrund der Art der Daten (öffentlich zugängliche Krankenhausqualitätsberichte und Befragung von Institutionen) war kein Ethikvotum erforderlich. Die statistische Analyse wurde mit χ^2^- und t‑Test durchgeführt, wobei ein Signifikanzniveau von *p* < 0,05 festgelegt wurde. Da nicht alle Fragebögen komplett beantwortet wurden, kann die Gesamtzahl variieren und ist in den Tabellen bei der Variablen angegeben. Alle Berechnungen wurden mit „IBM SPSS Statistics 28“ (Armonk, NY, USA) durchgeführt.

## Ergebnisse

### Kollektiv der urologischen Kliniken

Insgesamt zeigt sich in der Kohorte eine Rücklaufquote von 80 % (206/259 Kliniken). Hiervon sind 19 % (39/205) Universitätskliniken und 32 % (66/204) zertifizierte Kontinenzzentren. 37 % (76/205) der Kliniken haben bis 30 Betten, 46 % (95/205) 31–50 Betten und 17 % (35/205) > 50 Betten (Tab. 1).

**Tab. 1:** Kollektiv der deutschen urologischen Kliniken, die urodynamischer Studien (UDS) durchführen (*n* = 206)

		Gesamt (*n* = 206 [%])
Größe des Krankenhauses (*n* = 205)	Uniklinik	39 (19)
	Fachkrankenhaus	7 (3)
	Maximalkrankenhaus	52 (26)
	Zentralkrankenhaus	31 (15)
	Regelkrankenhaus	50 (24)
	Grundkrankenhaus	23 (11)
	Mindestkrankenhaus	3 (2)
Bettenzahl (*n* = 205)	1–10 Betten	5 (2)
	11–30 Betten	71 (35)
	31–50 Betten	95 (46)
	>50 Betten	35 (17)
Kontinenzzentrum (*n* = 204)	Ja	66 (32)
	Nein	138 (67)
Wer führt die Urodynamik durch?	ÄrztIn	191 (93)
	Pflege	93 (45)
	Urotherapeut	30 (15)
Wer wertet die Urodynamik aus? (*n* = 204)	Jede/r ÄrztIn	33 (16)
	Spezialisierte/r ÄrztIn	164 (81)
	Interdisziplinär	7 (3)
Welche Urodynamikuntersuchungen führen sie durch? (*n* = 205)	Uroflow	202 (99)
	Urethradruck	143 (70)
	Zystomanometrie	201 (98)
	Videourodynamik	109 (53)
Zahl der Urodynamikplätze (*n* = 205)	1	194 (94,5)
	2	8 (4)
	3	1 (0,5)
	4	2 (1)
Zahl der Urodynamiken pro Jahr (*n* = 197)	1–50	49 (25)
	51–100	65 (33)
	101–250	52 (26)
	251–500	24 (12)
	> 500	7 (4)
Verteilung der Indikationen (*n* = 189)	Unkomplizierte weibliche Belastungsinkontinenz	10,6 ± 12,7
		5 (0–70) %
	Komplizierte weibliche Belastungsinkontinenz	15,7 ± 13,1
		10 (0–90) %
	Rezidivinkontinenz	9,5 ± 8,3
		10 (0–70) %
	Deszensus der Frau	7,3 ± 8,2
		5 (0–50) %
	Überaktive Harnblase	28,3 ± 17,6
		25 (0–90) %
	Neurogene Blasenentleerungsstörung	21,4 ± 18,2
		20 (0–90) %
	Postoperative Blasenentleerungsstörung	9,9 ± 12,9
		5 (0–100) %
Hochrechnung der absoluten Verteilung der Indikationen (*n* = 189)	Unkomplizierte weibliche Belastungsinkontinenz	329,7 (7)
	Komplizierte weibliche Belastungsinkontinenz	867,3 (19)
	Rezidivinkontinenz	523,3 (12)
	Deszensus der Frau	281 (6)
	Überaktive Harnblase	923,2 (21)
	Neurogene Blasenentleerungsstörung	1080,9 (24)
	Postoperative Blasenentleerungsstörung	486,1 (11)

### Versorgungsrealität von Urodynamiken in urologischen Kliniken

**Abb. 1:**
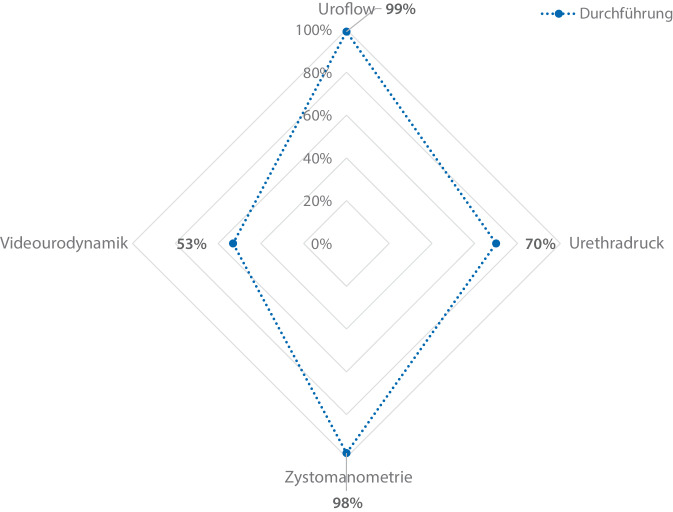
Überblick über die angewandten urodynamischen Untersuchungsverfahren in urologischen Kliniken (*n* = 205)

**Abb. 2:**
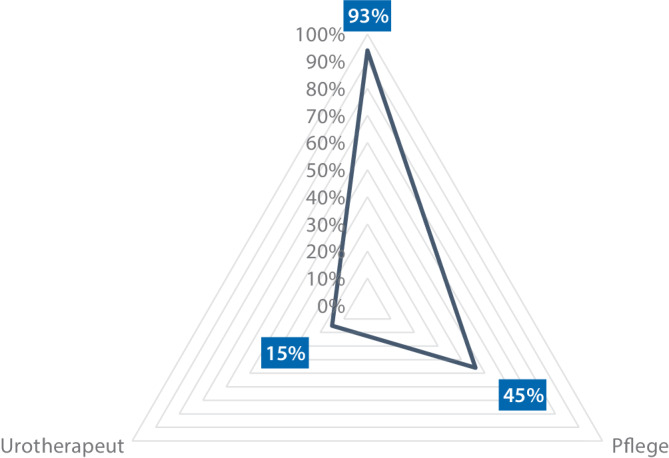
Durchführung urodynamischer Untersuchungen in urologischen Kliniken (*n* = 206)

**Abb. 3:**
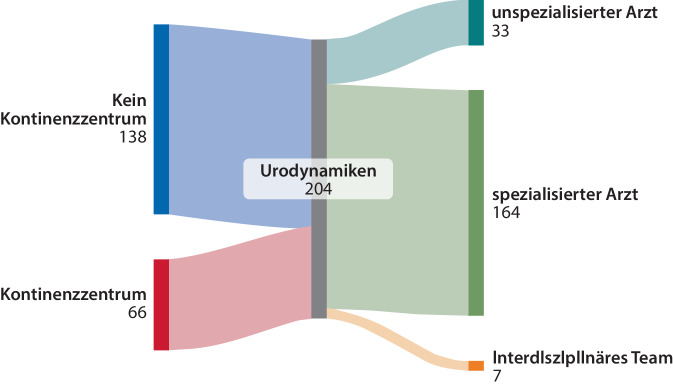
Auswertung von urodynamischen Untersuchungen in urologischen Kliniken (*n* = 204)

Die Ergebnisse spiegeln die aktuelle Versorgungsrealität urologischer Kliniken wider: 58 % (*n* = 114/197) der Kliniken führen bis zu 100 UDS/Jahr durch, während 42 % (*n* = 83/197) eine höhere Fallzahl (> 100 UDS/Jahr) aufweisen. Während Uroflowmetrien (99 % [*n* = 202/205]) und Zystomanometrien (98 % [*n* = 201/205]) nahezu flächendeckend etabliert sind, werden Urethradruckprofilmessungen nur in 70 % (*n* = 143/205) und Videourodynamiken in lediglich 53 % (*n* = 109/205) der Kliniken durchgeführt (Abb. 1). 2/205 (1 %) Kliniken führen nur Uroflowmetrien durch ohne Angabe invasiver urodynamischer Untersuchungen. Die Durchführung der UDS erfolgt in 93 % (*n* = 191/206) der Kliniken durch ärztliches Personal, jedoch übernehmen in 45 % (*n* = 93/206) auch Pflegekräfte und in 15 % (*n* = 30/206) spezialisierte Urotherapeuten diese Aufgabe (Abb. 2). In fast der Hälfte der Kliniken (48 %, [*n* = 98/206]) liegt die UDS-Durchführung ausschließlich in ärztlicher Hand. Die Auswertung der urodynamischen Ergebnisse erfolgt in 81 % (*n* = 164/204) der Kliniken durch einen spezialisierten Arzt, in 16 % (*n* = 33/204) durch einen beliebigen Arzt und in 3 % (*n* = 7/2024) interdisziplinär (Abb. 3; Tab. 1).

#### Verteilung der Indikationen in urologischen Kliniken

Die Kliniken geben im Durchschnitt folgende prozentuale Verteilung ihrer UDS-Indikationen an: unkomplizierte Belastungsinkontinenz der Frau: 10,6 ± 12,7 %, komplizierte Belastungsinkontinenz der Frau: 15,7 ± 13,1 %, Rezidivinkontinenz: 9,5 ± 8,3 %, Deszensus der Frau: 7,3 ± 8,2 %, überaktive Harnblase: 28,3 ± 17,6 %, neurogene Blasenfunktionsstörung: 21,4 ± 18,2 %, postoperative Blasenfunktionsstörung: 9,9 ± 12,9 %. Die Hochrechnung zeigt die folgende relative Verteilung der Indikationsstellungen: neurogene Blasenfunktionsstörung (24 %), überaktive Harnblase (21 %), komplizierte weibliche Belastungsinkontinenz (19 %), Rezidivinkontinenz (12 %), postoperative Blasenfunktionsstörung (11 %), unkomplizierte weibliche Belastungsinkontinenz (7 %), Deszensus der Frau (6 %) (Tab. 1). In Hinblick auf die Indikationsstellung von UDS bei der weiblichen Belastungsinkontinenz geben 57 % (*n* = 109/191) der Kliniken an, UDS sowohl bei komplizierter als auch unkomplizierter weiblicher Belastungsinkontinenz durchzuführen (Tab. 1).

Die Verteilung der Indikationen zur Durchführung einer UDS hängt teilweise von klinikspezifischen Parametern ab. Zertifizierte Kontinenzzentren und Universitätskliniken sowie Kliniken mit > 30 Betten führen signifikant häufiger UDS bei komplexeren Fällen wie neurogener Blasenentleerungsstörung durch (Universitätsklinikum: 30,3 ± 18,1 vs. 18,7 ± 16,6; Kontinenzzentrum: 22,7 ± 17,3 vs. 20 ± 7,5; Klinikgröße 21,5 ± 19,9 vs. 20,4 ± 15,8; Tab. 2). Zudem spielt der Anteil der zuweisenden Ärzte eine Rolle: Kliniken mit einem höheren Anteil an Zuweisern (> 25 %) führen insgesamt mehr UDS durch, insbesondere signifikant häufiger bei der überaktiven Harnblase (29,8 ± 16,9 vs. 23,6 ± 16,8; Tab. 2).

**Tab. 2:** Einfluss von Parametern auf die Indikationsverteilungen von urodynamischen Studien (UDS) in urologischen Kliniken

	Klinikgröße (Bettenzahl)			Universitätsklinikum			Kontinenzzentrum			Anteil Zuweiser			Zahl der Urodynamiken/Jahr		
Unkomplizierte weibliche Inkontinenz	≤ 30	12,9 ± 16	0,2	Ja	6,2 ± 8,4	*0,002*	Ja	9,1 ± 13	0,2	≤ 25 %	12,0 ± 13,8	0,5	≤ 250	11,1 ± 13,3	0,8
	(*n* = 66)	5 (0–70)		(*n* = 31)	5 (0–30)		(*n* = 54)	5 (0–70)		(*n* = 48)	10 (0–70)		(*n* = 150)	5 (0–70)	
	> 30	9,9 ± 10,5		Nein	12,1 ± 13,3		Nein	11,9 ± 12,7		> 25 %	10,6 ± 12,5		> 250	10,5 ± 10,5	
	(*n* = 114)	10 (0–45)		(*n* = 148)	10 (0–70)		(*n* = 125)	10 (0–70)		(*n* = 127)	5 (0–70)		(*n* = 25)	10 (0–30)	
Komplizierte weibliche Inkontinenz	≤ 30	14,5 ± 13,3	0,4	Ja	8,8 ± 6,4	*<* *0,001*	Ja	13,7 ± 9,5	0,3	≤ 25 %	14,8 ± 14,7	0,6	≤ 250	15,9 ± 13,4	0,3
	(*n* = 68)	10 (0–70)		(*n* = 32)	10 (0–20)		(*n* = 54)	10 (0–50)		(*n* = 50)	10 (0–90)		(*n* = 153)	10 (0–90)	
	> 30	16,0 ± 12,4		Nein	16,7 ± 13,2		Nein	15,9 ± 13,3		> 25 %	15,8 ± 12,1		> 250	12,8 ± 7,8	
	(*n* = 115)	15 (0–90)		(*n* = 150)	15 (0–90)		(*n* = 128)	15 (0–90)		(*n* = 178)	12,5 (0–70)		(*n* = 25)	15 (0–30)	
Rezidivinkontinenz	≤ 30	8,3 ± 6,6	0,2	Ja	10,4 ± 5,9	0,2	Ja	9,5 ± 6,6	0,6	≤ 25 %	8,6 ± 6,7	0,6	≤ 250	8,9 ± 6,7	0,4
	(*n* = 68)	5 (0–20)		(*n* = 30)	10 (0–20)		(*n* = 54)	10 (0–20)		(*n* = 49)	10 (0–20)		(*n* = 153)	10 (0–25)	
	> 30	9,5 ± 6,6		Nein	8,9 ± 6,8		Nein	8,9 ± 6,7		> 25 %	9,2 ± 6,6		> 250	10,2 ± 6,0	
	(*n* = 113)	10 (0–25)		(*n* = 150)	10 (0–25)		(*n* = 126)	10 (0–25)		(*n* = 176)	10 (0–25)		(*n* = 23)	10 (0–20)	
Deszensus	≤ 30	6,0 ± 8,2	0,5	Ja	5,8 ± 5,2	0,1	Ja	7,1 ± 7,2	0,8	≤ 25 %	7,8 ± 8,9	0,6	≤ 250	7,2 ± 7,7	0,7
	(*n* = 67)	5 (0–40)		(*n* = 29)	5 (0–20)		(*n* = 54)	5 (0–30)		(*n* = 48)	5 (0–40)		(*n* = 152)	5 (0–40)	
	> 30	7,6 ± 7,3		Nein	7,7 ± 8		Nein	7,4 ± 7,9		> 25 %	7,2 ± 7,3		> 250	7,7 ± 7,2	
	(*n* = 113)	5 (0–30)		(*n* = 150)	5 (0–40)		(*n* = 124)	5 (0–40)		(*n* = 127)	5 (0–30)		(*n* = 23)	5 (0–20)	
Überaktive Harnblase	≤ 30	28,1 ± 17,5	0,9	Ja	27,0 ± 13,6	0,3	Ja	31,2 ± 18,7	0,2	≤ 25 %	23,6 ± 16,8	*0,03*	≤ 250	29,4 ± 17,4	*<* *0,001*
	(*n* = 68)	25 (0–80)		(*n* = 33)	30 (5–60)		(*n* = 54)	30 (0–90)		(*n* = 50)	20 (0–80)		(*n* = 154)	28 (0–90)	
	> 30	28,5 ± 17,5		Nein	29,0 ± 18,2		Nein	27,2 ± 16,9		> 25 %	29,8 ± 16,9		> 250	19,4 ± 11,2	
	(*n* = 116)	25 (0–90)		(*n* = 150)	25 (0–90)		(*n* = 128)	25 (0–80)		(*n* = 129)	30 (0–90)		(*n* = 25)	20 (0–45)	
Neurogene Blasenfunktionsstörung	≤ 30	21,5 ± 19,9	0,7	Ja	30,3 ± 18,1	*<* *0,001*	Ja	22,7 ± 17,3	0,3	≤ 25 %	20,6 ± 19,5	0,8	≤ 250	19,7 ± 15,8	*0,04*
	(*n* = 68)	15 (0–90)		(*n* = 32)	25 (10–90)		(*n* = 55)	20 (0–70)		(*n* = 50)	10 (0–90)		(*n* = 154)	20 (0–90)	
	> 30	20,4 ± 15,8		Nein	18,7 ± 16,6		Nein	20 ± 17,5		> 25 %	21,4 ± 16,9		> 250	30,4 ± 24,1	
	(*n* = 116)	20 (0–80)		(*n* = 151)	10 (0–90)		(*n* = 127)	20 (0–90)		(*n* = 129)	20 (0–90)		(*n* = 25)	20 (5–90)	
Postoperative Blasenfunktionsstörung	≤ 30	9,8 ± 17,0	0,8	Ja	13,3 ± 10	*0,03*	Ja	9,4 ± 7,5	0,9	≤ 25 %	14,9 ± 19,9	*0,009*	≤ 250	9,4 ± 12,7	0,6
	(*n* = 66)	5 (0–100)		(*n* = 33)	10 (0–50)		(*n* = 54)	10 (0–30)		(*n* = 46)	10 (0–100)		(*n* = 150)	5 (0–100)	
	> 30	9,5 ± 7,7		Nein	8,8 ± 12,2		Nein	9,6 ± 13,5		> 25 %	7,6 ± 6,6		> 250	10,9 ± 6,8	
	(*n* = 114)	10 (0–30)		(*n* = 146)	5 (0–100)		(*n* = 124)	5 (0–100)		(*n* = 129)	5 (0–30)		(*n* = 25)	10 (2–30)	

Die kursiv gestellten Werte sind die signifikanten Werte

### Versorgungsrealität im ambulanten Sektor

Den Fragebogen beantworteten 44 % (122/280) der ambulanten UrologInnen . Von diesen führten 15 % (18/122) UDS durch. Die Versorgungsrealität im Praxisalltag zeigt, dass die UDS durch die UrologInnen (59 % (10/17)) oder die Arzthelferin (41 % [7/17]) durchgeführt werden und seltener durch eine Urotherapeutin (12 % [2/17]) oder einer Mitarbeiterin des UDS-Geräteanbieters (6 % [1/17]). Urethradruckprofile werden in 67 % (12/18), Flow-EMG in 61 % (11/18), Druck-Fluss-Messungen in 56 % (10/18), Provokationsmessungen in 22 % (4/18) und Videourodynamik in 11 % (2/18) der Praxen mit UDS durchgeführt (Tab. 3).

**Tab. 3:** Kollektiv urologischer Praxen (*n* = 122)

		Gesamt (*n* = 122 [%])
Praxisgröße (*n* = 119)	Einzelpraxis	38 (32)
	Gemeinschaftspraxis	25 (21)
	MVZ	56 (47)
Patienten pro Quartal (*n* = 121)	< 500	5 (4)
	500–1000	21 (17)
	1001–2000	68 (57)
	> 2000	27 (22)
Führt die Praxis selbst UDS durch?	Nein	104 (85)
	Ja	18 (15)
Wer führt die UDS durch? (*n* = 17)	ÄrztIn	10 (59)
	ArtzthelferIn	7 (41)
	UrotherapeutIn	2 (12)
	MitarbeiterIn des Urodynamikanbieters	1 (6)
Welche UDS führen sie durch? (*n* = 18)	Uroflow	16 (89)
	Urethradruckprofil	12 (67)
	Zystomanometrie	16 (89)
	Flow-EMG	11 (61)
	Druck-Fluss-Messung	10 (56)
	Provokationstests (z. B. Eiswassertest)	4 (22)
	Videourodynamik	2 (11)
Anteil weibliche Belastungsinkontinenz (*n* = 105)		23,5 ± 22,3
		15 (0–90) %
Anteil männliche Belastungsinkontinenz (*n* = 105)		6,1 ± 7,3
		5 (0–35) %
Anteil LUTS (*n* = 105)		11,3 ± 14,2
		10 (0–70) %
Anteil überaktive Harnblase (*n* = 105)		26,4 ± 17,2
		20 (0–80) %
Anteil neurogene Blasenfunktionsstörung (*n* = 105)		30,3 ± 23,9
		25 (0–90) %

*UDS* urodynamische Studien, *LUTS* „lower urinary tract symptoms“, *MVZ* medizinisches Versorgungszentrum, *EMG* Elektromyographie

### Verteilung der Indikationen in urologischen Praxen

Die Praxen geben im Durchschnitt folgende prozentuale Verteilung ihrer UD-Indikationen an: Belastungsinkontinenz der Frau: 23,5 ± 22,3 %, Belastungsinkontinenz des Mannes: 6,1 ± 7,3 %, LUTS des Mannes 11,3 ± 14,2 %, überaktive Harnblase: 26,4 ± 17,2 % und neurogene Blasenfunktionsstörung: 30,3 ± 23,9 % (Tab. 3).

## Diskussion

Die UDS sind nach wie vor von großer Bedeutung für die Diagnostik urologischer Erkrankungen, werden jedoch weniger häufig durchgeführt [16–18]. Es zeigt sich in urologischen Kliniken und Praxen insgesamt eine breite Indikationsverteilung für UDS. Die Verteilung der Indikationen an Kliniken variiert in Abhängigkeit spezifischer Klinikparameter.

In der praktischen Umsetzung von Urodynamiken in urologischen Kliniken zeigt sich, dass die Durchführung von UDS derem technischen Aufwand entspricht. Uroflowmetrien (99 %) und Zystomanometrien (98 %) führen fast alle Kliniken durch. Urethradruckprofilmessungen werden ebenfalls häufiger angeboten (70 %), während die Videourodynamik mit dem zusätzlich notwendigen Röntgenarbeitsplatz nur in etwa der Hälfte der Kliniken möglich ist (53 %). Obwohl 93 % der Kliniken angeben, dass UDS auch von ÄrztInnen durchgeführt werden, werden in 48 % der Kliniken die ÄrztInnen als alleinige durchführende Personen genannt. In den übrigen Kliniken scheinen Pflegekräfte an der Durchführung beteiligt zu sein, was auf eine mögliche Aufgabenverteilung hindeutet – beispielsweise mit einer Vorbereitung durch Pflegekräfte und der eigentlichen Untersuchung durch ÄrztInnen. Eine genauere Differenzierung der spezifischen Aufgaben erfolgte in der Befragung jedoch nicht.

In 15 % der Kliniken werden UDS sogar durch spezialisierte UrotherapeutInnen durchgeführt. Die stärkere Einbindung von spezialisierten Pflegekräften könnte aus ärztlicher Sicht eine sinnvolle Entlastung darstellen. Um dies zu fördern, wenden sich Urodynamikkurse wie die des Arbeitskreises „Urologische Funktionsdiagnostik und Urologie der Frau“ gezielt auch an Pflegekräfte. Dennoch sollten auch ÄrztInnen in ihrer Ausbildung praktische Erfahrung in der UDS-Durchführung sammeln, um ein besseres Verständnis für die Methodik und Interpretation der Ergebnisse zu entwickeln.

Angesichts des Fachkräftemangels sowohl in der Pflege als auch im ärztlichen Bereich wird die tatsächliche Aufgabenverteilung vermutlich je nach lokaler Situation unterschiedlich ausfallen [19]. Die Auswertung der UDS-Ergebnisse spiegelt die Komplexität des Verfahrens wider, da sie überwiegend durch spezialisierte Ärzte durchgeführt wird (81 %). Hier stellt sich die Frage, inwieweit AssistenzärztInnen im Rahmen ihrer Facharztausbildung in die Auswertungen mit eingebunden werden oder damit gar nicht in Kontakt kommen [20].

Insgesamt zeigen die Ergebnisse, dass trotz einer weitgehenden Standardisierung der urodynamischen Verfahren Unterschiede in der praktischen Durchführung und Befundung bestehen. Diese hängen maßgeblich von der jeweiligen Klinikstruktur und den personellen Ressourcen ab. Insbesondere die ungleiche Verteilung der durchführenden Berufsgruppen sowie die limitierte Verfügbarkeit komplexerer Verfahren wie der Videourodynamik unterstreichen, dass die Versorgungsrealität stark von strukturellen Gegebenheiten abhängt.

Die häufigsten Indikationen zur Durchführung einer UDS sind die neurogene Blasenentleerungsstörung (24 %) und die überaktive Harnblase (21 %). Dies unterstreicht die Relevanz der UDS bei diesen Krankheitsbildern. Laut Leitlinie ist die Urodynamik die Diagnostik der Wahl bei neurogener Blasenentleerungsstörung und bei der überaktiven Harnblase nach erfolglosen konservativen Therapieversuch [21, 22]. Postoperative Blasenentleerungsstörungen (11 %) und Rezidivinkontinenz (12 %) sind ebenfalls häufige Indikationen.

Interessanterweise nimmt die weibliche Kontinenzproblematik auch einen wesentlichen Anteil der durchgeführten UDS in deutschen Kliniken ein. Die Rezidivinkontinenz (12 %) und die komplizierte Belastungsinkontinenz (19 %) spielen eine bedeutende Rolle bei der Indikationsverteilung. Aber auch bei der unkomplizierten weiblichen Belastungsinkontinenz werden UDS durchgeführt (7 %). Dies scheint zunächst im Widerspruch zur Leitlinienempfehlung zu stehen, nach der bei unkomplizierter weiblicher Belastungsinkontinenz keine Indikation zur UDS besteht [11, 23–25]. In einer multizentrischen, randomisierten Studie von Nager et al. konnte gezeigt werden, dass eine UDS bei Frauen mit unkomplizierter Stressinkontinenz keinen signifikanten Einfluss auf das Gesamtergebnis nach Inkontinenzoperationen hatte [11, 26]. Ebenso konnte die multizentrische Studie von van Leijsen et al. zeigen, dass der Verzicht auf UDS bei der präoperativen Abklärung von Frauen mit Belastungsinkontinenz nicht weniger wirksam war als ihr Einsatz [27, 28]. Trotz dieser Evidenz zeigen unsere Ergebnisse, dass UDS weiterhin für diese Indikation durchgeführt werden. Dies könnte auf Unsicherheiten in der praktischen Umsetzung der Leitlinien zurückzuführen sein oder darauf, dass in bestimmten Fällen ergänzende diagnostische Maßnahmen für notwendig erachtet werden. Limitierend kann hier nicht ausgeschlossen werden, dass Kliniken, die diese Indikation angegeben haben, sich nur auf die Durchführung von Urethradruckprofilen beziehen und nicht auf eine Zystomanometrie.

Weiterhin zeigen die Ergebnisse, dass die Indikationsverteilung der UDS maßgeblich von verschiedenen Parametern abhängt (Tab. 2). Neurogene Blasenfunktionsstörungen werden häufiger in Universitätskliniken bzw. Kliniken mit einer hohen UDS-Fallzahl durchgeführt. Dies unterstreicht die Komplexität dieses Krankheitsbildes und die damit zu befürwortende Versorgung an spezialisierten Zentren, die viele UDS durchführen. Insbesondere bei Universitätskliniken zeigt sich eine Selektion dieser Indikationen. Hier werden häufiger postoperative Blasenfunktionsstörungen und neurogene Blasenfunktionsstörungen versorgt. Im Gegensatz dazu spielen UDS im Rahmen von Harninkontinenz eine untergeordnete Rolle an Universitätskliniken. Hier ist zu vermuten, dass die Inkontinenzversorgung sich schwerpunktmäßig eher auf nichtuniversitäre Kliniken verteilt.

Die Indikation der überaktiven Harnblase wird häufig in Kliniken mit einem hohen Anteil an externen Zuweisern und einer hohen UDS-Fallzahl durchgeführt. Dies unterstreicht zum einen die hohe Prävalenz der überaktiven Harnblase und zum anderen die Bedeutung dieses Krankheitsbildes im ambulanten Bereich [29].

Die Indikationsverteilung in Praxen zeigt ein ähnliches Bild wie in Kliniken. Den Hauptteil nehmen die neurogene Blasenfunktionsstörung und die überaktive Harnblase ein. Da nur 15 % der Praxen UDS durchführen, wird der Hauptteil dieser Indikationen in den Kliniken durchgeführt. Die Verteilung der unterschiedlichen UDS spiegelt auch hier den Aufwand wider. Insbesondere Videourodynamiken wurden nur in 2/15 Praxen durchgeführt, was den hohen Aufwand mit einer Röntgenanlage widerspiegelt (Tab. 3). Betrachtet man den zeitlichen und personellen Aufwand für eine UDS, so steht dies nicht im Verhältnis zur Vergütung (EBM 26313: 102,03 €; [30]). Insofern lässt sich nachvollziehen, dass die Zahl an UDS in Praxen abnimmt und heutzutage eher eine Ausnahme ist [10, 15]. Auch in Kliniken könnte die geringe Vergütung ein zusätzlicher Faktor sein, der zur sinkenden Zahl an UDS beiträgt, da der wirtschaftliche Druck auf Krankenhäuser zunehmend wächst und kosteneffiziente Diagnostik bevorzugt wird.

Eine Limitation der Studie ist die Datenerhebung mittels Fragebögen, insbesondere, da die Angaben zur Indikationsverteilung auf Schätzungen der Kliniken basieren. Die absoluten Verteilungen der Indikationen stellen daher Hochrechnungen dar, die aus den geschätzten Anteilen und der Gesamtzahl der UDS in den jeweiligen Kliniken abgeleitet wurden. Weiterhin sind die Indikationen nicht weiter detailliert aufgeschlüsselt nach der Art neurogener Blasenentleerungsstörungen oder der Differenzierung der operativen Eingriffe bei postoperativen Blasenentleerungsstörungen. Es wurden nur die Indikationsstellungen für UDS in urologischen Abteilungen analysiert, während gynäkologische und pädiatrische Abteilungen sowie Rehabilitationszentren nicht berücksichtigt wurden. Unsere Daten stammen aus dem Jahr 2023. Entsprechend unserer vorherigen Studie macht der Anteil an UDS in der Gynäkologie nur 4 % aller UDS aus [10, 15]. Der Anteil an UDS für weibliche Inkontinenz wird in gynäkologischen Abteilungen vermutlich höher sein in Relation zum Anteil urologischer Kliniken mit 33 %. Auch Rehabilitationszentren führen einen großen Teil an UDS durch (> 30 %; [10, 15]). Hier wird die Indikation der neurogenen Blasenfunktionsstörung im Vordergrund stehen. Somit spiegelt die vorliegende Studie nur die Versorgungswirklichkeit in urologischen Kliniken und Praxen wider. Darüber hinaus stellt die Erhebung der UDS-Versorgung in ambulanten Praxen aufgrund der allgemein niedrigen Rücklaufquote eine Herausforderung dar. Um diesem Problem zu begegnen, wurde eine repräsentative Stichprobe herangezogen. Mit einer Rücklaufquote von 44 % übertrifft diese zwar die durchschnittlichen Werte in ähnlichen Erhebungen, erreicht jedoch nicht das Niveau der Kliniken, wodurch potenzielle Verzerrungen dennoch reduziert werden konnten.

**Abb. 4:**
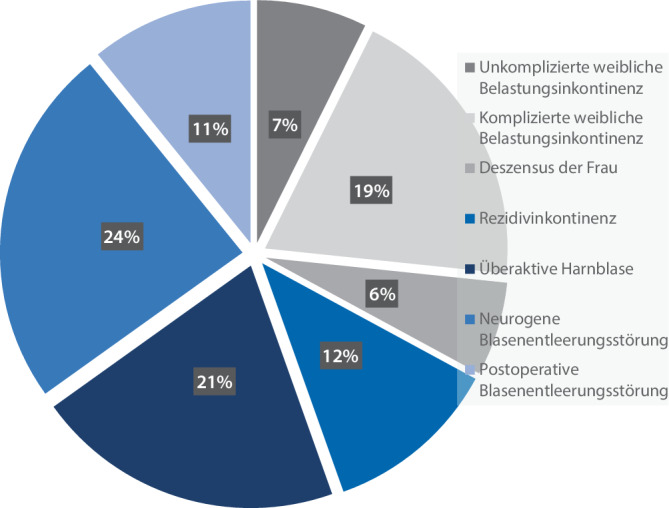
Überblick über die Verteilung der Indikationsstellungen von Urodynamiken in urologischen Kliniken (*n* = 189)

Nichtsdestotrotz liefert diese Studie erstmals einen Überblick zur Nutzung und Indikationsstellung von UDS in deutschen urologischen Kliniken und Praxen. Mit einer Rücklaufquote von 80 % der urologischen Abteilungen wird ein präzises Abbild der UDS-Versorgungsrealität im urologischen Klinikalltag präsentiert. Die differenzierte Verteilung der Indikationen spiegelt sowohl die Vielseitigkeit der UDS als auch ihre Relevanz für spezifische Krankheitsbilder wider und bietet eine Grundlage für zukünftige Diskussionen zur Weiterentwicklung dieser Diagnostik in Deutschland (Abb. 4; [11, 31]).

## Fazit für die Praxis

- Es zeigt sich eine breite Indikationsverteilung für die Durchführung von urodynamischen Studien (UDS) in deutschen urologischen Kliniken und Praxen.
- Die neurogene Blasenentleerungsstörung und die überaktive Harnblase sind die häufigsten Indikationen zur Durchführung einer UDS.
- Die Indikationsverteilung kann sich an Kliniken unterscheiden – abhängig von der Klinik, Fallzahl und Zuweiserstruktur.

## Data Availability

Die in dieser Studie verwendeten Daten sind auf begründete Anfrage beim entsprechenden Autor erhältlich.
